# Inhibition of Na^+^/K^+^-ATPase induces hybrid cell death and enhanced sensitivity to chemotherapy in human glioblastoma cells

**DOI:** 10.1186/1471-2407-14-716

**Published:** 2014-09-26

**Authors:** Dongdong Chen, Mingke Song, Osama Mohamad, Shan Ping Yu

**Affiliations:** Department of Anesthesiology, Emory University School of Medicine, Atlanta, GA 30322 USA; Department of Hematology and Oncology, Emory University School of Medicine, 101 Woodruff Circle, Suite 620 Woodruff Memorial Research Building, Atlanta, GA 30322 USA

**Keywords:** Na^+^ pump, Glioblastomas, Apoptosis, Hybrid cell death, K^+^ homeostasis, Intracellular Ca^2+^, Temozolomide

## Abstract

**Background:**

Glioblastoma multiforme (GBM) is very difficult to treat with conventional anti-cancer/anti-apoptotic drugs. We tested the hypothesis that inhibition of Na^+^/K^+^-ATPase causes a mixed or hybrid form of concurrent apoptosis and necrosis and therefore should enhance anti-cancer effects of chemotherapy on glioblastoma cells.

**Methods:**

In human LN229 and drug-resistant T98G glioblastoma cell cultures, cell death and signal pathways were measured using immunocytochemistry and Western blotting. Fluorescent dyes were applied to measure intracellular Ca^2+^, Na^+^ and K^+^ changes.

**Results:**

The specific Na^+^/K^+^-ATPase blocker ouabain (0.1 - 10 μM) induced cell death and disruption of K^+^ homeostasis in a time- and concentration-dependent manner. Annexin-V translocation and caspase-3 activation indicated an apoptotic component in ouabain cytoxicity, which was accompanied with reduced Bcl-2 expression and mitochondrial membrane potential. Ouabain-induced cell death was partially attenuated by the caspase inhibitor Z-VAD (100 μM). Consistently, the K^+^ ionophore valinomycin initiated apoptosis in LN229 cells in a K^+^ efflux-dependent manner. Ouabain caused an initial cell swell, which was followed by a sustained cell volume decrease. Electron microscopy revealed ultrastructural features of both apoptotic and necrotic alterations in the same cells. Finally, human T98G glioblastoma cells that are resistant to the chemotherapy drug temozolomide (TMZ) showed a unique high expression of the Na^+^/K^+^-ATPase α2 and α3 subunits compared to the TMZ-sensitive cell line LN229 and normal human astrocytes. At low concentrations, ouabain selectively killed T98G cells. Knocking down the α3 subunit sensitized T98G cells to TMZ and caused more cell death.

**Conclusion:**

This study suggests that inhibition of Na^+^/K^+^-ATPase triggers hybrid cell death and serves as an underlying mechanism for an enhanced chemotherapy effect on glioblastoma cells.

**Electronic supplementary material:**

The online version of this article (doi:10.1186/1471-2407-14-716) contains supplementary material, which is available to authorized users.

## Background

Glioblastoma multiforme (GBM), also called glioblastoma, is the most common and aggressive primary brain tumor in adults. It is classified as a Grade IV brain tumor according to the World Health Organization (WHO) classification. Due to its aggressive biological behavior, diffuse infiltrative growth and central location, it has become one of the most challenging cancers of the central nervous system (CNS) [1, 2]. The therapeutic approach to glioblastoma includes maximal safe resection surgery followed by radiation therapy plus concomitant and adjuvant chemotherapy [1, 3]. Glioblastoma often show little to no response to conventional anti-cancer drugs such as temozolomide (TMZ) and it becomes resistant to apoptosis after a short period of treatment. This is especially true for invasive malignant glioma cells that are resistant to pro-apoptotic chemotherapy and radiotherapy [4–6]. The current 5-year overall survival of grade IV GBM patients using radiotherapy with concomitant TMZ treatment is only 9.8%. The resistance to chemotherapy remains a critical issue in the failure of successful treatment of cancer, especially in GBM patients.

Tumor-induced hypoxic barriers, existence of cancer stem cells, enhanced membrane transporter activities and other mechanisms may be important factors in drug resistance [7]. One way in which cancer cells can achieve resistance to anti-cancer drugs is by up-regulating the ATP-binding cassette transporter proteins which are responsible for the efflux of anti-cancer molecules from the intracellular compartment [8]. Another mechanism of resistance to chemotherapy involves the hypoxic conditions in the central portions of the tumor and the resultant over-expression of HIF-1α that enhances a cell’s tolerance to insults including anti-cancer drugs. Furthermore, hypoxic cells may be less proliferative and thus less responsive to anti-cancer drugs that target rapidly proliferating cells [9]. We hypothesize that a new therapeutic approach that can simultaneously trigger more than one cell death program/mechanism may have a better chance of overcoming the drug resistance of glioblastoma cells.

Na^+^/K^+^-ATPase, also known as the Na^+^ pump or more accurately the Na^+^/K^+^ pump, is a ubiquitously expressed transmembrane transporter composed of tetramers of alpha and beta subunits. A normal activity of Na^+^/K^+^-ATPase is essential for maintaining ionic homeostasis, cellular pH, and cell volume [10]. The catalytic alpha subunit is a large polypeptide of ~1,000 amino acid residues, which catalyzes the ion-dependent ATPase activity and carries the binding sites for ATP and the specific inhibitor ouabain. The beta subunit is a smaller polypeptide of about 300 residues, which regulates conformational stability and activity of the alpha subunit. The Na^+^/K^+^ pump is critical in maintaining high extracellular Na^+^ (~145 mM) and high intracellular K^+^ (~150 mM) by pumping Na^+^ ions out of the cell and importing K^+^ ions into the cell [11]. By doing so, these Na^+^/K^+^ pumps maintain a physiological electrochemical gradient that is essential for cell survival and for many cellular activities. Consistent with its pro-life role, Na^+^/K^+^-ATPase is highly expressed in cancer cells including glioblastoma cells [12–14]. The Na^+^/K^+^ pump activity increases during the course of malignant cell transformation [15]. This increased expression and elevated activity suggest that Na^+^/K^+^-ATPase may serve as a biological marker and a therapeutic target of cancer cells. Along with the identification of its high expression in cancer cells and its critical roles in cell survival, proliferation, adhesion and migration, the clinical potential of Na^+^/K^+^-ATPase modulators such as cardiotonic steroids or digitalis in oncology has drawn increasing attention in recent years [12, 16]. Several cardenolides have been shown to display *in vitro* antitumor activities against various types of cancer cells [17–21], including glioma cells [22, 23].

Cardiac glycosides including digoxin, marinobufagenin, telocinobufagin and ouabain, represent a group of compounds isolated from plants and animals [24]. Endogenous ouabain-like substances were also identified as a hormone or stress signal that responds to exogenous and endogenous stimuli such as physical exercise, stress, hypertension, hypoxia/ischemia, among many others [24]. These cardiac glycosides have been used in clinical therapies of heart failure and atrial arrhythmia for many years [19, 24]. Meanwhile, digoxin acts as a specific neuroblastoma growth inhibitor in mice grafted with the neuroblastoma cell lines SH-SY5Y and Neuro-2a [25]. Blocking Na^+^/K^+^-ATPase using the exogenous cardiac glycoside ouabain is cytotoxic to a variety of cancer and non-cancerous cells; the sensitivity depends on the expression level of the functional Na^+^/K^+^ pump and dosage used [26–29]. Ouabain and the specific knockdown of the Na^+^/K^+^-ATPase alpha subunit inhibits cancer cell proliferation and migration [13, 22], sensitizes resistant cancer cells to anoikis and decreases tumor metastasis [30]. However, the cellular/molecular mechanisms underlying the cytotoxic effect of cardiac glycosides in tumor cells have been poorly defined. We noticed that blocking Na^+^/K^+^-ATPase has two direct and marked impacts on the cellular ionic homeostasis: increased intracellular Na^+^ concentration and decreased intracellular K^+^ concentration. The majority of previous studies have been focused on the intracellular Na^+^ increase and the consequent intracellular Ca^2+^ increases due to the enhanced reversal operation of the Na^+^-Ca^2+^ exchanger [31–33]. On the other hand, increasing evidence from our groups and other’s have demonstrated that, in many noncancerous neuronal and non-neuronal cells, depletion of intracellular K^+^ is a prerequisite for apoptotic cell shrinkage, activation of caspases and initiation of apoptotic programing [34–36]. Consistently, attenuating the outward K^+^ current with tetraethylammonium or elevating extracellular K^+^ prevented apoptosis while treatment with the K^+^ ionophore valinomycin induced apoptosis [37, 38], There is also evidence that cytosolic Ca^2+^ levels may not directly regulate apoptotic cell death [11, 39]. Therefore, besides the regulation by a series of apoptotic genes, apoptosis is regulated by an ionic mechanism closely associated with K^+^ homeostasis [11, 39, 40]. Up to now, little attention has been paid to the intracellular K^+^ loss in cancer cells.

We previously demonstrated in different noncancerous cells that inhibition of Na^+^/K^+^-ATPase induced a mixed form of cell death composed of concurrent necrotic and apoptotic components in the same cells, which we named hybrid death [41]. Specifically, the increases in intracellular Na^+^ and Ca^2+^ are associated with necrosis and K^+^ depletion is linked to apoptosis. These events may take place simultaneously and trigger activation of multiple signaling pathways. The identification of hybrid cell death was also based on cellular/sub-cellular morphological changes, gene expression, and alterations in intracellular signaling pathways [11, 41].

In this investigation, we tested the main hypothesis that inhibition of Na^+^/K^+^-ATPase could disrupt K^+^ and Na^+^/Ca^2+^ homeostasis and subsequently induce hybrid death in human glioblastoma cells. Ouabain was tested because of its high selectivity in blocking NA^+^/K^+^-ATPase. We also tested whether inhibition of Na^+^/K^+^-ATPase or deletion of its specific subunit could enhance the sensitivity of glioblastoma cells to TMZ in the drug-resistant T98G glioblastoma cells.

## Methods

### Cultures of human glioblastoma cells

Human glioblastoma cell lines LN229 and T98G (kindly supplied by Dr. Erwin G. Van Meir, Emory University, Winship Cancer Institute) were maintained in Dulbecco’s modified Eagle’s media supplemented with 10% fetal bovine serum (FBS).

### Ethics statement

LN229 and T98G cells are established cell lines from glioblastoma of anonymous patients and are commercially available. These cells have been extensively used in cancer research and related information is publically available. Therefore, their use was not classified as human subject research, and no Institutional Review Board approval was needed.

### Cell viability assay by MTT spectrophotometry

Cells were cultured at a density of 3000 cells/well in 96-well plates at 5% CO_2_ and 37 °C. At 70% confluence, cells were treated with either ouabain or other drugs. At selected time points, 3-(4,5-dimethyl-thiazol-2-yl)-2,5-diphenyltetrazolium (MTT) was added at a final concentration of 0.5 mg/mL. After 4 hrs incubation, the reaction was stopped by adding a solubilization buffer (10% SDS, 10 μM HCl). After the mixture was incubated at 37°C for 2 hrs, the relative optical density for each well was determined at 570 nm by a microplate spectrophotometer (Bio-Tek, Winooski, Vermont).

### Apoptosis detection by flow cytometry

Phosphatidylserine (PS) membrane translocation and caspase-3 activation were determined by flow cytometry using FITC Annexin V Apoptosis Detection Kit (BD Pharmingen, San Diego, CA). Cells were treated with 1 μM ouabain or 10 μM valinomycin for selected time points and then washed twice with phosphate-buffered saline (PBS). Staining procedures followed the standard protocol provided by the manufacturer. Briefly, 1 × 10^6^ cells were resuspended in 1 mL of binding buffer and then the 100 μL cell suspension was incubated with 1 μL Annexin-V-FITC and 1 μL propidium iodide (PI) for 15 min at room temperature in the dark. Propidium iodide was used as a marker of necrosis. The population of Annexin V-positive cells was evaluated by a BD Biosciences LSR II flow cytometer and analyzed by FlowJo Version 7.6 software (Tree Star, Ashland, OR).

### Western blotting analysis

Cells were lysed in protein lysis buffer (25 mM Tris–HCl (pH 7.4), 150 mM NaCl, 5 mM EDTA, 0.1% SDS, 2 mM sodium orthovanadate, 100 mM NaF, 1% Triton, leupeptin, aprotinin, and pepstatin) containing protease inhibitor (Sigma, St Louis, MO). Protein concentration was determined using the Bicinchoninic Acid Assay (Sigma). 30 μg protein samples were separated by SDS-polyacrylamide gel electrophoresis (SDS-PAGE) in a Hoefer Mini-Gel system (Amersham Biosciences, Piscataway, NJ) and transferred onto a PVDF membrane (BioRad, Hercules, CA). The blotting membrane was incubated with primary antibodies overnight: Bcl-2 and cleaved Caspase-3 (1:1000, Cell Signaling, Danvers, MA), Cytochrome c and Caspase-9 (1:500, Millipore, Billerica, MA), β-actin (1:5000, Sigma). The blots were incubated for 1 hr at room temperature with anti-mouse or anti-rabbit alkaline phosphatase-conjugated IgG antibodies (1:2000, Promega, Madison, WI). The signals were detected by the addition of 5-bromo-4-chloro-3-indolylphosphate/nitroblue tetrazolium (BCIP/NBT) solution (Sigma) and quantified and analyzed by the NIH imaging software Image J (NIH, Bethesda, MD). The level of protein expression was normalized to β-actin controls. The value of protein levels was designed as 1 in the control group. The results were expressed as mean proportion of the control group values.

### Immunocytochemistry staining

Cells were fixed with 4% paraformaldehyde and then treated with 0.2% Triton-X 100 for 5 min. After blocking with 1% fish gel for 1 hr, cells were incubated with primary anti-body AIF overnight (1:500, Millipore). Cells were then incubated with secondary antibody Cy3-conjugated anti-rabbit IgG (1:500, Invitrogen, Carlsbad, CA) for 1 hr at room temperature. Nuclei were stained with Hoechst 33342 (1:20000, Invitrogen). Staining was visualized by fluorescent and confocal microscopy (BX61; Olympus, Japan).

### Fluorescent measurement of the mitochondrial membrane potential

Cells were treated with ouabain or valinomycin for 6 hrs and then loaded with 200 nM TMRM (Molecular Probes, Eugene, OR) for 30 min at 5% CO_2_ and 37°C in the dark. Prior to imaging, cells were washed with DMEM medium twice. Fluorescent images were captured by a fluorescent microscope (Leica DMIRB, Germany) and fluorescent intensity was measured by the NIH imaging software Image J.

### Cellular ion measurements

Intracellular K^+^ content was measured using the cell permeant potassium indicator PBFI-AM (Invitrogen, Molecular Probes). Cells were washed with HBSS and then loaded with 5 μM PBFI and 10 μM F-127 for 40 min at 5% CO_2_ and 37°C in the dark. Cells were washed with HBSS three times before imaging. Measurements were made by exciting PBFI at 340 nm while monitoring emission at 500 nm using a fluorescent microscope (Leica DMIRB, Germany) and the fluorescence intensity was measured using the NIH imaging software Image J.

Intracellular Na^+^ content was measured using the cell permeant sodium indicator SBFI-AM (Invitrogen, Molecular Probes). Cells were washed with HBSS and then loaded with 5 μM SBFI and 10 μM F-127 for 40 min at 5% CO_2_ and 37 °C in the dark. After three HBSS washes, fluorescent imaging was carried out at room temperature using an inverted fluorescence microscope (Olympus IX81, Olympus America Inc., Center Valley, PA). Measurements were made by exciting SBFI at 340 nm while monitoring emission at 520 nm using a CCD camera. The imaging data were recorded with a digital camera Hamamatsu ORCA-ER (Hamamatsu Photonics K.K., Japan) and software Slidebook 4.1 for Windows (SciTech Pty Ltd., Australia).

Intracellular free Ca^2+^ was measured using the cell permeate Ca^2+^ sensitive dye Fluo-4-AM (Invitrogen; 5 μM in 100 μl HEPES buffered solution) for 50 min at 5% CO_2_ and 37°C in the dark. Fluo-4 epifluorescence was excited at 480 nm light and images were obtained at 520 nm. The imaging data were collected by the same fluorescence microscopy system described for sodium imaging.

### Cell volume assay

Cells were trypsinized after drug treatments. A 100 μL cell suspension of each sample was taken by Millipore Scepter™ Handheld Automated cell counter (Millipore). Cell volume was measured and analyzed by Scepter Software Pro 2.0.

### Electron microscopic examination of ultrastructural changes

Cultures in 35 mm dishes were fixed in glutaraldehyde (1% glutaraldehyde, 0.1 M sodium cacodylate buffer, pH 7.4) for 30 min at 4 °C, washed with 0.1 M sodium cacodylate buffer, and post-fixed in 1.25% osmium tetroxide for 30 min. The staining and electron microscopy was performed at the Robert P. Apkarian Integrated Electron Microscopy Core (Emory University, Atlanta, GA).

### Cytochrome c release assay

Cells were harvested by centrifugation at 200 g for 10 min at 4°C. Mitochondrial and cytoplasmic proteins were isolated using the Mitochondria Isolation Kit (Thermo Scientific, Rockford, IL) according to the kit’s instructions. Cytochrome c released from the mitochondria was detected by Western blot.

### Knockdown of the Na^+^/K^+^-ATPase α3 subunit

Na^+^/K^+^-ATPase α3 stealth RNAi™ siRNA duplex oligoribonucleotides were synthesized by Invitrogen. The sequences of the siRNA duplex were designed by Invitrogen Block-iT RNAi Designer:

Forward: 5′-ACG ACA ACC GAU ACC UGC UGG UGA U-3′

Reverse: 5′-AUC ACC AGC AGG UAU CGG UUG UCG U-3′

The T98G cells were transfected with Na^+^/K^+^-ATPase α3 stealth RNAi™ siRNA or stealth RNAi™ siRNA negative control (Invitrogen) using Lipofectamine™ 2000 (Invitrogen) according to the manufacture’s instruction. Briefly, 0.5 × 10^5^ T98G cells were plated in a 6-well plate and cultured overnight. 250 pmol siRNA duplex or siRNA negative control was mixed with 10 μL lipofectamine reagent in the serum free Opti-MEM medium and transfected the T98G cells for 6 hrs. 48 hrs later, the cells were harvested for the reverse transcriptase-polymerase chain reaction (PCR) to detect the expression of the α3 subunit.

### Reverse transcriptase-polymerase chain reaction

Total RNA was extracted from human glioblastoma cells using the Trizolreagent (Invitrogen) according to the procedure suggested by the manufacturer. For cDNA synthesis, 1 μg of total RNA were reverse transcribed into cDNA using RNA to cDNA High Capacity kit (Invitrogen) and PCR was performed in a PTC-150 Minicycler (MJ Research Inc., Watertown, MA) with primer sets for target genes and a housekeeping gene, ribosomal protein large subunit 19 (RPL19) as an internal control for both cDNA quantity and quality. PCR primers, as listed below, were designed according to the sequences in a previous report [42]. All the primers were designed to amplify products that covered one or more exons.

Na^+^/K^+^-ATPase á1 forward 5′-GAA AGA AGT TTC TAT GGA TG-3′

reverse 5′-ATG ATT ACA ACG GCT GAT AG-3′

Na^+^/K^+^-ATPase á2 forward 5′-AGA GAA TGG GGG CGG CAA GAA G-3′

reverse 5′-TGG TTC ATC CTC CAT GGC AGC C-3′

Na^+^/K^+^-ATPase á3 forward 5′-CCT CAC TCA GAA CCG CAT GAC-3′

reverse 5′-TTC ATC ACC AGC AGG TAT CGG-3′

RPL19 forward 5′-GAG TAT GCT CAG GCT TCA GA-3

reverse 5′-TTC CTT GGT CTT AGA CCT GC-3′

After an initial phase at 94°C for 2 min, amplification of α1 was run for 31 cycles and α 2 and α 3 for 40 cycles. The cycles consisted of denaturation at 94°C for 1 min, annealing at 50°C for 45 s for α 1 and 54°C for 1 min for α 2 and α 3, extension at 72°C for 1 min, and a final extension of 7 min at 72°C at the end of the program. The PCR products (25 μL) in TAE buffer were loaded onto 1.5% agarose gel and run at 36 V for 90 min. The Gel was scanned for quantitative analysis using the UnScan It program (Silk Scientific Inc., Orem, UT). The ratio of target gene to housekeeping gene, RPL19, was calculated.

### Chemicals

The caspase inhibitor Z-VAD-FMK was purchased from Enzyme Systems Products [42]. BAPTA-AM was from Tocris Bioscience (Bristol, UK). Ouabain and valinomycin were from Sigma Aldrich (St. Louis, MO) [42].

### Statistical analyses

One-way ANOVA followed by Tukey post-test was performed for multiple group comparisons. Two-way ANOVA followed by Bonfferoni post-tests was used for multiple groups with multiple time points. Data were shown as mean ± SEM. Changes were identified as significant if *p* value was less than 0.05.

## Results

### Ouabain-induced cell volume changes and toxicity in LN229 cells

**Figure 1:**
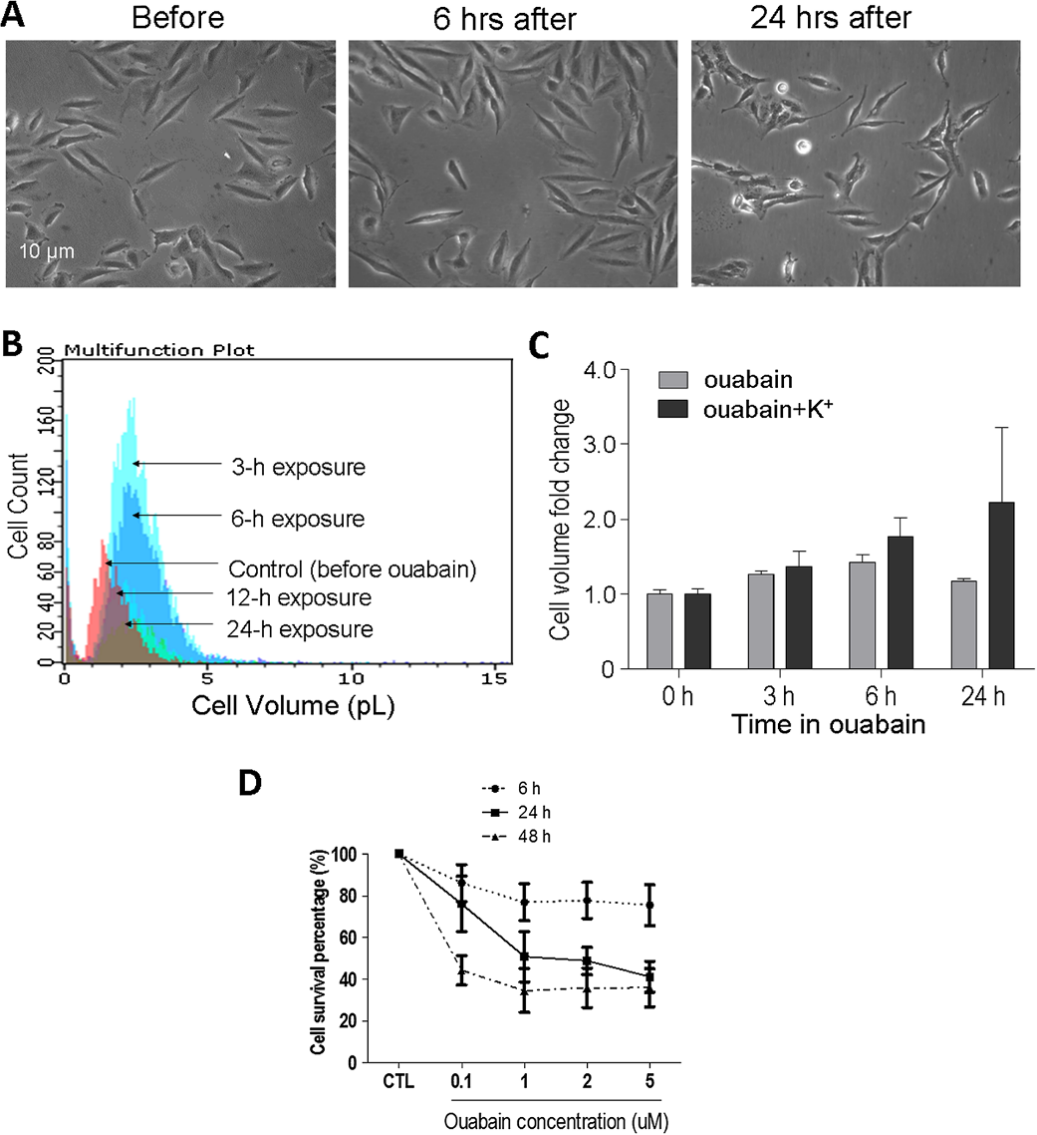
**Ouabain-induced cell volume changes and cytotoxicity in LN229 cells** ***.*** Oubain-induced cell volume changes and toxicity were inspected in LN229 cell cultures to delineate the time- and concentration-dependent consequences of blocking Na^+^/K^+^-ATPase. **A**. Phase contrast images showing morphological changes of LN229 cells during 6 to 24 hrs exposures to ouabain (1 μM). **B**. Cell volume distributions examined using a Millipore ScepterTM Handheld Automated cell counter illustrated a dynamic cell volume regulation during 24-hr exposure to ouabain (1 μM). A transient but noticeable cell swelling was seen at 3 and 6 hrs after ouabain treatment, while the cell volume returned to the original size after 12 to 12 hrs in ouabain. **C**. Cell volume changes were assessed and compared between ouabain (1 uM) in normal medium containing 5 mM K^+^ and ouabain exposure in an elevated K^+^ medium (25 mM KCl). Although cells exposed to ouabain in normal medium returned to their original sizes after 24-hr exposure, the ouabain exposure in the high K^+^ medium eliminated the cell shrinking phase. **D**. The time- and concentration-dependent cytotoxic effects of ouabain in LN229 cultures. Cell viability was measured using the MTT assay. In general, the longer the exposure time, the lower cell viability was induced by ouabain. Increasing ouabain concentration from 0.1 μM to 1 μM induced further reduction in cell viability. Even higher ouabain concentrations (2 and 5 μM) showed no further increase in toxic effect. Three independent experiments and each group in an experiment contained 3–4 duplicates.

Exposure of glioblastoma LN229 cells to ouabain caused noticeable morphological changes including cell swelling and, as a sign of membrane deterioration, granule structures started to appear on the surface of the cell membrane (Figure 1A). A quantified analysis revealed that the cell swelling developed soon after exposure to ouabain (1 μM) and reached the peak around 3–6 hrs later (Figure 1B-C). Interestingly, the swollen cells gradually returned to the original size regardless of the continuous presence of ouabain in the medium (Figure 1B-C). Moreover, raising the extracellular K^+^ concentration from 5 to 25 mM showed no effect on cell swelling but prevented the belated cell volume reduction, implying that a K^+^ efflux mechanism mediated the cell volume decrease (Figure 1C).

As an initial test for ouabain induced cytotoxicity in human glioblastoma cells, we exposed LN229 cultures to different ouabain concentrations. The MTT assay showed that ouabain induced time- and concentration-dependent cell viability reduction in these cells (Figure 1D). At a low concentration of 0.1 μM that is sublethal to normal neuronal cells [43], ouabain caused 13.8%, 23.9% and 42.0% reduction in cell viability after 6-, 24- and 48-hr exposures, respectively. Increasing ouabain concentration from 0.1 to 1 μM significantly augmented the cytotoxic effect at all time points (Figure 1D). Even higher concentrations (2 and 5 μM) did not further increase the toxicity at 24 to 48 hrs (Figure 1D). In the following experiments, 1 μM ouabain was selected to produce toxic effect in LN229 cells.

### Ouabain-induced activation of apoptotic cascade in LN229 cells

**Figure 2:**
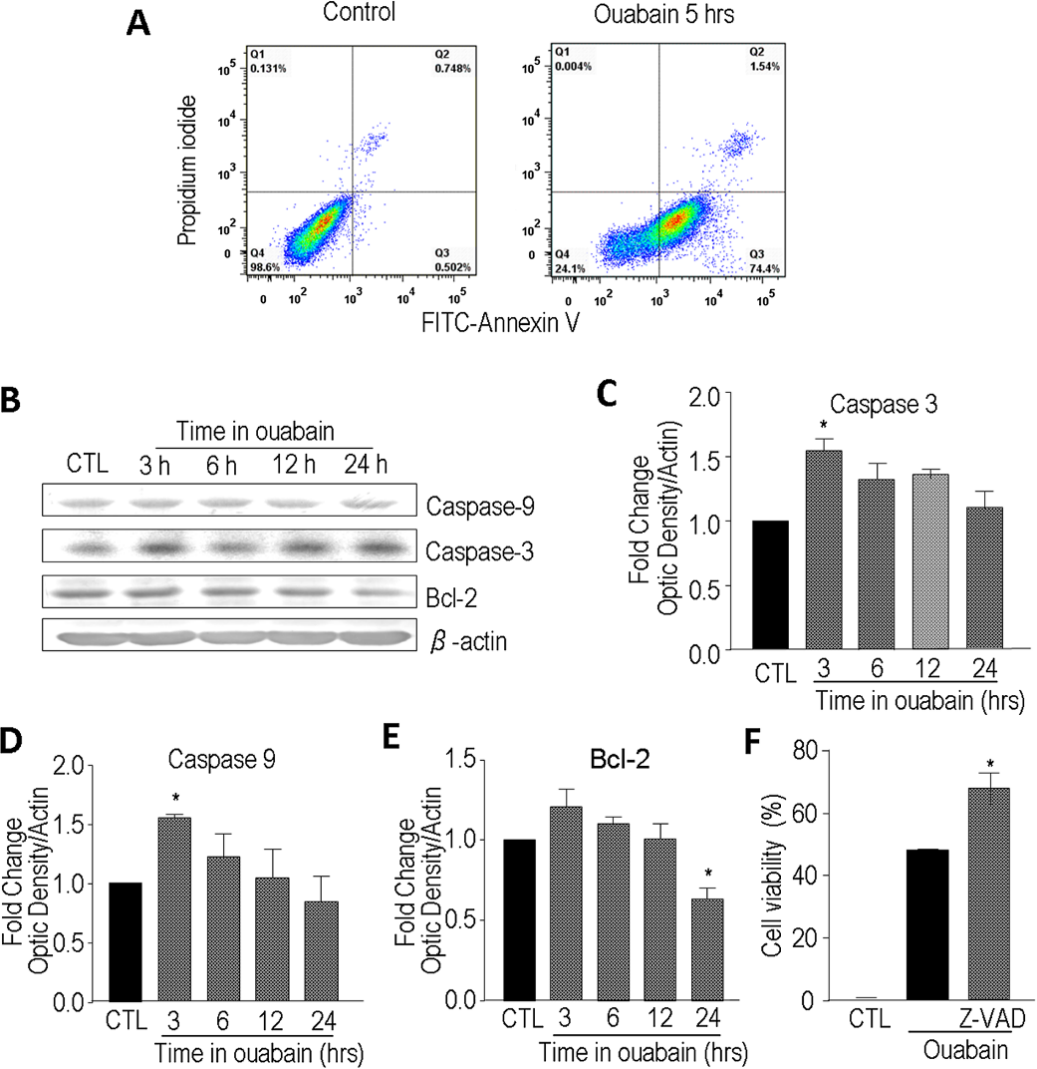
**Ouabain-induced caspase-dependent apoptosis in LN229 cells.** Ouabain-induced cell death and apoptotic signaling were evaluated using flow cytometry and Western blot analysis. **A**. Flow cytometry results showed that ouabain treatment (1 μM, 5 hrs) increased early (Q3 quadrant) and late apoptotic (Q2 quadrants) cell populations. N = 3 independent assays. **B**, **C**, and **D**. Western blot analysis revealed that ouabain treatment of 3 hrs induced significant activation of caspase-3 and caspase-9 in LN229 cells. The caspase activations subsided thereafter. **E**. Western blotting indicated that although the anti-apoptotic gene Bcl-2 showed a trend of increasing at an early stage during ouabain exposure, the Bcl-2 level significantly decreased 24 hs after ouabain treatment. N = 3 in each group. **F**. Ouabain-induced cell death in LN229 cells was partially blocked by co-applied caspase inhibitor Z-VAD (100 uM). Cell death was measured using the MTT assay. Three independent experiments and each group in an experiment contained 3–4 duplicates. * *P* < 0.05 vs. control (CTL).

The translocation of phosphatidylserine (PS) from the cytoplasmic side of the plasma lipid membrane to the membrane outer surface is an early event in apoptosis. Annexin V has been widely used as a probe for detecting this PS translocation event. Ouabain-induced PS translocation in LN229 cells was inspected using flow cytometry. Annexin V-positive cells significantly increased after a 5-hr exposure to ouabain (1 μM) (Figure 2A). Meanwhile, ouabain treatment for 3 hrs significantly increased activation of caspase-3 and caspase-9 (Figure 2). The activation of caspases was also detected using Western blot analysis (Figure 2C to E). Maximal activation of both caspases-3 and caspase-9 occurred 3 hrs after ouabain treatment and then dropped to control levels after 24 hrs. The anti-apoptotic protein Bcl-2 expression was reduced 24 hrs after ouabain treatment (Figure 2F). In line with the apoptotic component in ouabain-induced death of LN229 cells, co-applied caspase pan inhibitor Z-VAD-FMK (100 μM) significantly attenuated the ouabain-induced cell death (Figure 2). There was a cell death component, however, that was not prevented by the high concentration of Z-VAD (IC_50_ ≤ 10 uM for caspase inhibition), suggesting that there were caspase-independent cell death mechanisms in ouabain cytotoxicity yet to be identified.

### Ouabain-induced loss of mitochondrial membrane potential in LN229 cells

**Figure 3:**
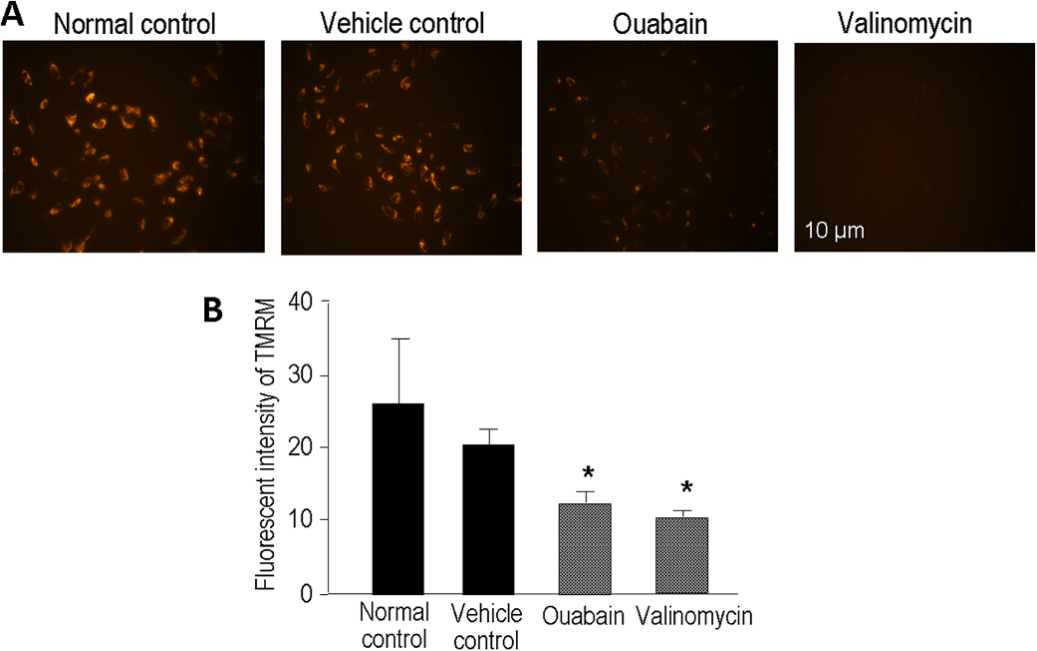
**Ouabain and valinomycin induced mitochondrial membrane depolarization in LN229 cells.** The mitochondrial membrane potential was assessed using the fluorescent dye TMRM in LN229 cells. **A**. TMRM (200 nM) was added into the medium to stain the live cells for 30 min. The intensity of TMRM fluorescence images is a reflection of the mitochondrial membrane potential. The reduction and disappearance of TMRM staining was seen 6 hrs after ouabain (1 μM) and valinomycin (10 μM) treatment. **B**. Quantification of TMRM fluorescence intensity after 6 hrs of ouabain treatment. Both ouabain and valinomycin induced a significant loss of the mitochondrial membrane potential in LN229 cells. DMSO was a vehicle negative control. The fluorescent intensity was quantified using the NIH Image J software. * *P* < 0.05 vs. controls.

The loss of mitochondrial membrane potential is an early event indicating dysfunction of energy metabolism and cell damage associated with both apoptosis and necrosis [44–47]. Tetramethylrhodamine methyl ester (TMRM) is a cell membrane permeable cationic dye that is actively sequestered by live mitochondria and has been used to detect changes in mitochondrial membrane potential [48]. In LN229 cells, 6-hr exposure to 1 μM ouabain caused a marked decrease in orange-red fluorescence of TMRM, indicating a significant loss of mitochondrial membrane potential and damage to the cells (Figure 3A and B).

### Valinomycin- and Ouabain-induced disruption in K^+^ homeostasis and its relation to apoptotic events

**Figure 4:**
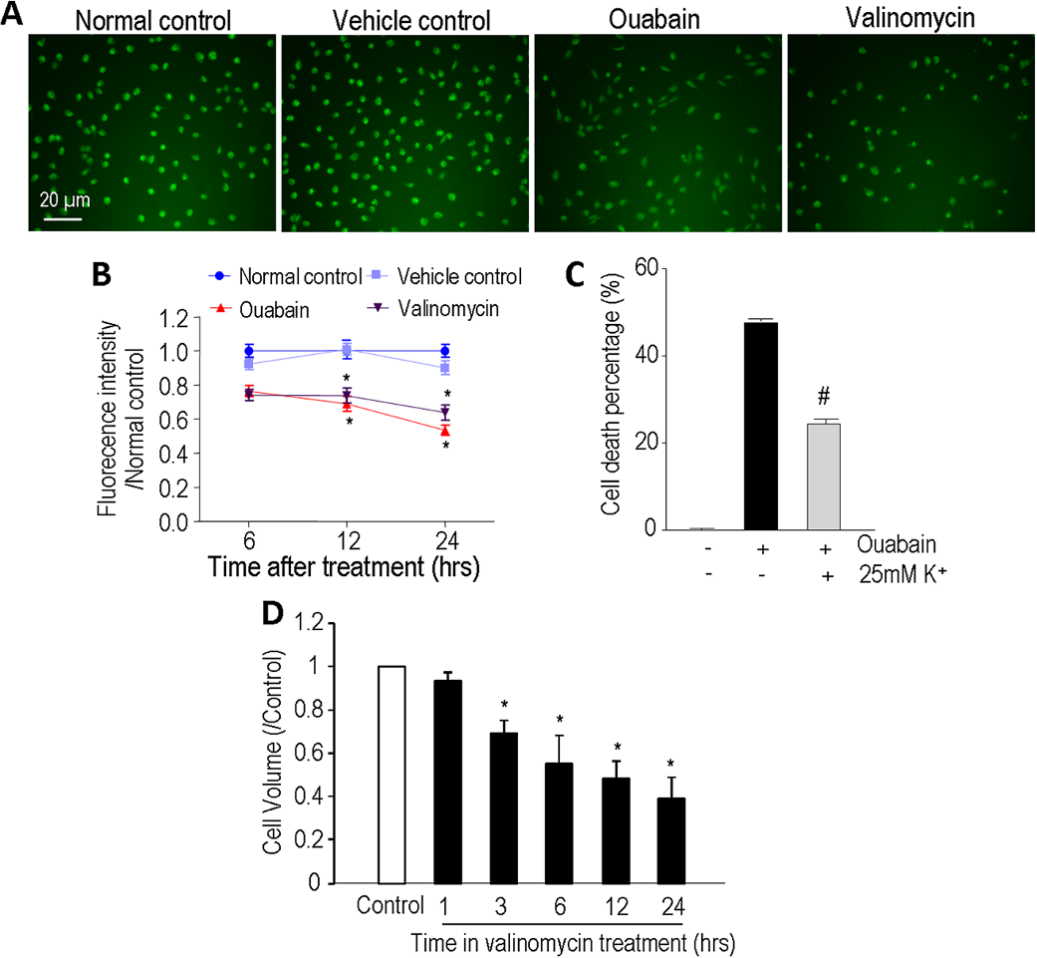
**Ouabain and valinomycin induced intracellular K** ^**+**^ **depletion in LN229 cells.** The K^+^ fluorescent dye PBFI-AM was used to measure the intracellular K^+^ changes of LN229 cells during ouabain and valinomycin exposures. **A**. PBFI images illustrated reduction in intracellular K^+^ content 24 hrs after ouabain (1 μM) or valinomycin (10 μM) treatment. **B**. Quantified data of PBFI imaging at 6, 12 and 24 hrs exposures to ouabain and valinomycin. By 24 hrs after exposure, ouabain and valinomycin each caused aproximatley 50% of cellular K^+^ loss. This could be equivalent to a loss of about 70 mM K^+^ from the intracellular space. DMSO was tested as the vehicle control. N = 180 cells from 3 independent assays in each group. * *P* < 0.05 vs. control groups. **C**. Cell death induced by ouabain was sensitive to a protective effect of high K^+^ extracellular medium. Around 50% of cell death was prevented in the high K^+^ medium. Cell death was measured using the MTT assay 24 hrs after exposure. The control medium contained 5 mM KCl. ^#^ *P* < 0.05 vs. ouabain group. **D**. Valinomycin (10 μM) induced cell shrinkage after 3, 6, 12, 24 hrs exposure in LN229 cells. * *P* < 0.05 vs. control group.

The disruption of K^+^ homeostasis has been linked to initiation of an apoptotic cascade in many non-cancer cells [11, 34–36, 39, 40]. To detect whether the K^+^-mediated mechanism might contribute to ouabain-induced cell death in human glioblastoma cells, we measured the intracellular K^+^ content using the cell permeable K^+^ indicator PBFI-AM. As a control, we first treated LN229 cells with the K^+^ ionophore valinomycin that is well known for its highly specific selectivity for K^+^ flux through lipid membranes down the K^+^ electrochemical gradient [38]. As expected, valinomycin (10 μM) induced a dramatic depletion of intracellular K^+^, significant loss of the mitochondrial membrane potential, and noticeable cell shrinkage in LN229 cells (Figure 4). The PBFI fluorescent intensity dropped significantly at 6 hrs after ouabain treatment and continued dropping at 12 and 24 hrs. Thus, ouabain treatment resulted in a marked and continuous depletion of intracellular K^+^ that lasted for many hours, leading to ~50% K^+^ loss by 24 hrs (Figure 4B). We confirmed that valinomycin increased caspase-3 activation after 6-, 12- and 24-hr exposure, while significant caspase-9 activation was seen at 12 hrs after valinomycin exposure (Additional file 1: Figure S1 A-C). Meanwhile, the anti-apoptotic protein Bcl-2 expression decreased at 12 and 24 hrs (Additional file 1: Figure S1 D). Valinomycin also stimulated a nuclear translocation of the Apoptosis-Inducing Factor (AIF), which represents a caspase-independent apoptotic pathway (Additional file 1: Figure S1 E). These tests verified that, similar to many non-cancerous neuronal and non-neuronal cells, glioblastoma cells are sensitive to the K^+^ efflux mediated apoptosis.

Supporting the idea that excessive K^+^ efflux is critical in apoptotic cell death, attenuating K^+^ efflux by elevating extracellular K^+^ to 25 mM antagonized ouabain-induced cell death (Figure 4C). To exclude the possibility that the effect of high K^+^ medium was mediated via membrane depolarization associated Ca^2+^ influx, additional experiments were performed in the presence of the Ca^2+^ channel blocker nifedipine (1 μM). This maneuver, however, did not eliminate the protective effect of the 25 mM K^+^ medium (data not shown, but see [11, 41]). Another important point is that, as in the case with Z-VAD, the high K^+^ medium only partially attenuated ouabain toxicity, confirming there were other injurious mechanisms in ouabain-induced cell death.

### Ouabain-induced intracellular Ca^2+^ and Na^+^ Changes in LN 229 Cells

It is widely accepted that necrosis is triggered by increases in intracellular Ca^2+^ and Na^+^, while blocking the Na^+^/K^+^ pump is expected to cause accumulation of intracellular Na^+^ and Ca^2+^. This effect, however, has not been verified in human glioblastoma cells before. We thus measured the intracellular Na^+^ and Ca^2+^ using the cell permeable indicators SBFI-AM and Fluo-4-AM, respectively. As expected, SBFI imaging showed that ouabain increased intracellular Na^+^ as early as 5 min after addition of ouabain and the effect lasted for up to one hour (Additional file 2: Figure S2 A and B). Fluo-4-AM Ca^2+^ imaging showed that intracellular Ca^2+^ concentration ([Ca^2+^]_i_) doubled after 3–6 hr treatment with 1 μM ouabain (Additional file 2: Figure S2 C and D). The [Ca^2+^]_i_ increase, however, subsided at 24 hrs after ouabain treatment. To determine the role of this [Ca^2+^]_i_ increase in ouabain-induced cytotoxicity, the membrane permeable Ca^2+^ chelator BAPTA-AM was added into the media to prevent the increase in [Ca^2+^]_i_. BAPTA-AM (1 μM) effectively prevented ouabain-induced [Ca^2+^]_i_ increases in LN229 cells (Additional file 2: Figure S2). However, addition of BAPTA-AM did not antagonize ouabain-induced cell death; rather it showed a trend of increasing ouabain-induced cell death in MTT assays. This was likely due to a toxic effect of BAPTA alone on the survival of LN229 cells (Additional file 2: Figure S2 E).

### Ouabain-induced ultrastructural changes of hybrid cell death in glioblastoma cells

**Figure 5:**
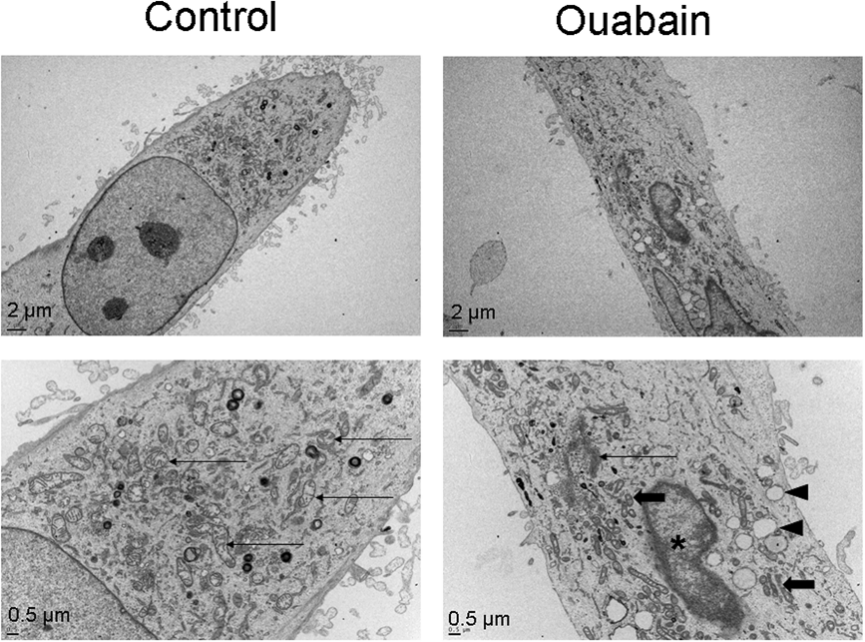
**Ouabain-induced ultrastructual features of hybrid cell death in LN229 cells.** Electron microscopy was applied to examine the ultrastructual features of ouabain damaged glioblastoma cells. The lower panel is magnified images. Control cells showed intact plasma membranes, intact mitochondrial (arrows) and other organelles. Ouabain exposure (24 hrs) resulted in apparent necrotic changes such as breakdown of the plasma membrane, cytoplasmic swelling, appearance of large and empty vacuoles (arrow head), which is possibly an indication of autophagic activity. There were also signs of apoptotic pathology such as fragmented and shrinking mitochondria surrounded by the intact membrane (thick arrows), and acondensed/fragmented nucleus (*). Note that there was nuclear shrinkage in the absence of cell shrinkage, which is another sign of hybrid cell death.

Since the morphological changes, especially ultrastructural ones, have been regarded as a gold standard for distinguishing apoptosis from necrosis, we used electron microscopy to examine the ultrastructural features of ouabain-induced cell death. Electron microscopy imaging revealed that ouabain treatment (1 μM, 24 hrs) caused breakdown of the plasma membrane, while the nucleus showed shrinkage in the absence of absolute cell volume decrease. Cytosol swelling accompanied the appearance of many empty vacuoles in the cytoplasm (Figure 5). These subcellular alterations are typical in cells dying from the hybrid cell death mechanism previously observed in non-cancerous cells [41, 43, 49].

### High expression of the Na^+^/K^+^-ATPase subunits in glioblastoma cells and its relation to resistance to TMZ

**Figure 6:**
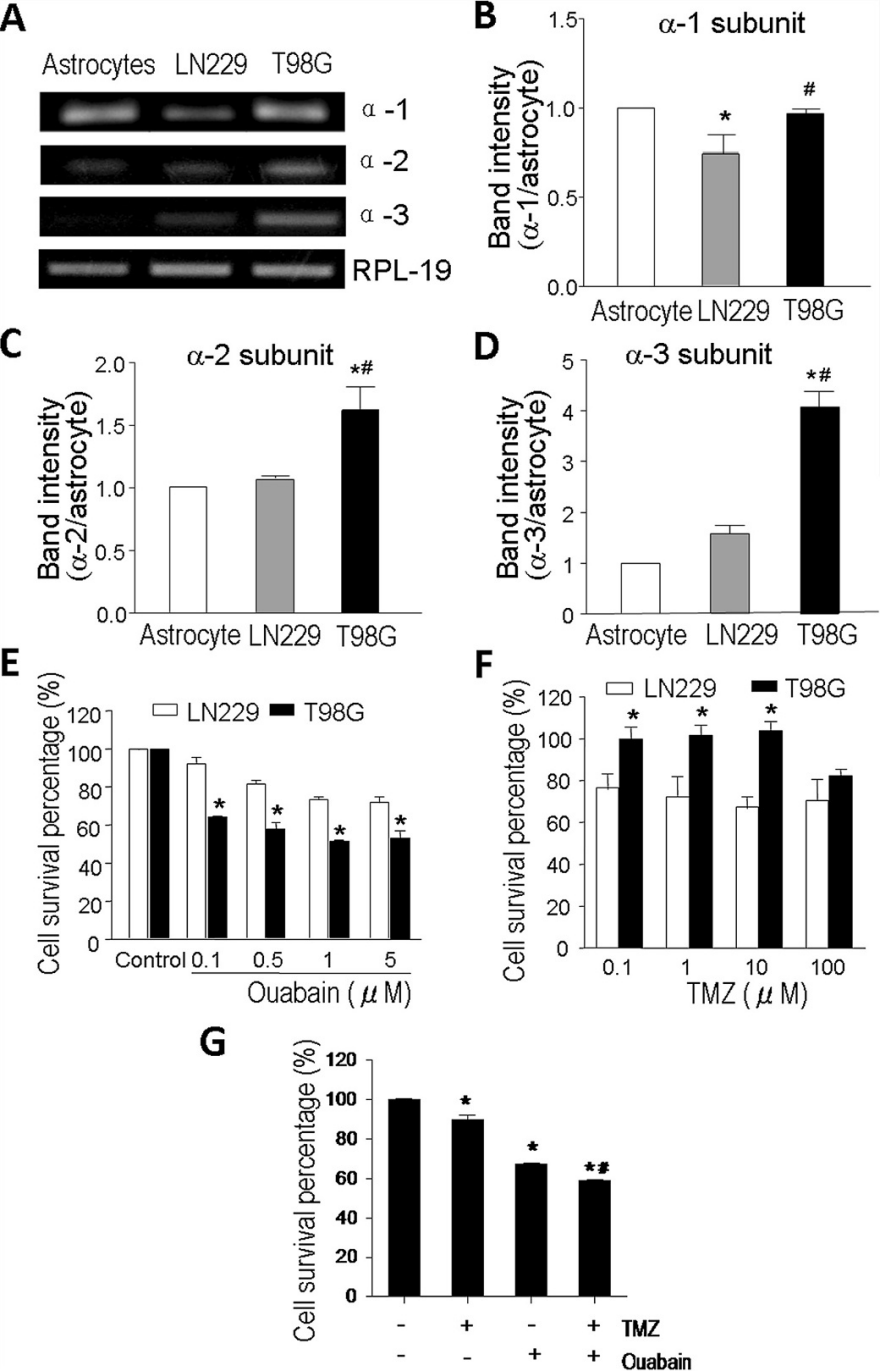
**Expression of Na** ^**+**^ **/K** ^**+**^ **-ATPase subunits in LN229/T98G cells and human astrocytes.** The expression of Na^+^/K^+^-ATPase three α subunits was detected using reverse transcriptase PCR analysis in glioblastoma cell lines LN229 and T98G as well as in human astrocytes. **A**. RT-PCR result shows different mRNA levels of α1, α2 and α3 subunits in the three cell types. Ribosomal protein-19 (RPL-19) was measured as a housekeeper gene for loading control. **B**. Quantified data in the bar graph show that T98G cells express higher α1 mRNA levels compared to LN229 cells but there is no difference in α1 mRNA levels compared to human astrocytes. N = 3 in each group; * *P* < 0.05 vs. human astrocytes; ^#^ *P* < 0.05 vs. LN229 cells. **C** and **D**. Quantified RT-PCR data on α2 **(C)** and α3 **(D)** subunits. Both subunits were highly expressed in T98G cells and were significant different from those in LN229 cells and human astrocytes. **E**. MTT assay of cell viability showed that ouabain treatment (0.1 - 5 μM for 6 hrs) significantly reduced the cell viability in T98G cells compared to LN229 cells. **F**. MTT assay confirmed a higher resistance of T98G cells to TMZ (0.01 – 100 μM for 24 hrs) compared to LN229 cells. ^*^ *P* < 0.05 vs. LN229 cells. **G**. MTT assay showed that co-treatment of ouabain (0.1 μM) and TMZ (100 μM) for 24 hrs significantly augmented the cell vulnerability to TMZ. ^*^ *P* < 0.05 vs. control, ^#^ *P* < 0.05 vs. TMZ alone.

In an effort to understand a possible relationship between Na^+^/K^+^-ATPase and high resistance to chemotherapy drugs, we examined the expression of Na^+^/K^+^-ATPase subunits α1, α2 and α3 in TMZ-sensitive LN229 cells, TMZ-resistant T98G cells, as well as normal human astrocytes. While T98G cells expressed more α1 mRNA compared to LN229, the expression of α1 mRNA was not statistically different from human astrocytes (Figure 6A and B). It was then interesting to see that T98G cells expressed higher mRNA levels of the α2 and α3 subunits compared to LN229 cells and human astrocytes. α3 subunit level was more than doubled in T98G cells compared to LN229 cells and 4 folds the level in human astrocytes (Figure 6A to D)

More importantly, T98G cells were more sensitive to ouabain-induced cell death (0.1 to 5 μM) (Figure 6E), and were more resistant to TMZ compared to LN229 cells (Figure 6F). It is worth pointing out that the low concentration of 0.1 μM ouabain does not affect the viability of LN229 cells, normal astrocytes and non-cancerous neuronal cells [43], while it showed a significant killing effect on T98G cells. Meanwhile, TMZ at low concentrations induced negligible cell death in T98G cells, which instead kept proliferating in the presence of low dose TMZ (Figure 6F). Only when the TMZ concentration was elevated to 100 μM did T98G cells show a very mild cell death response (Figure 6F). These data suggested a selective action of ouatain on TMZ-resistant tumor cells.

In the next experiment, we tested the TMZ killing effect on T98G and LN229 cells with and without Na^+^,K^+^-ATPase inhibition. At a relatively low dosage (0.1 μM), ouabain was coapplied with TMZ (100 μM). This co-application significantly augmented the death of T98G cells compared to TMZ treatment alone (Figure 6G). This data supported the idea that inhibition of the Na^+^/K^+^ pump activity with relatively low dosages of ouabain could increase the susceptibility of the drug-resistant T98G cells to TMZ.

### Knockdown of the Na^+^/K^+^-ATPase α3 subunit sensitizes drug resistant T98G cells to TMZ

**Figure 7:**
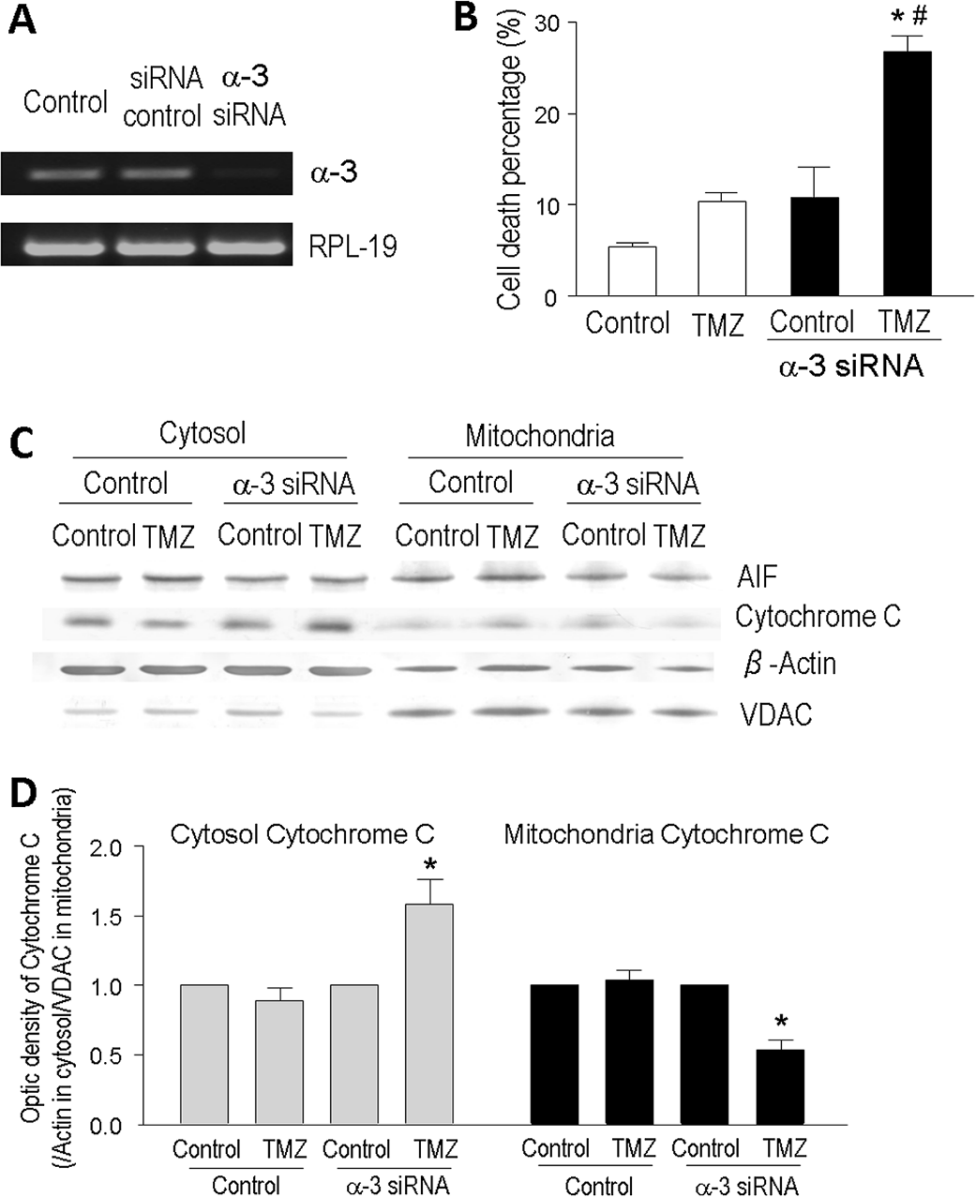
**Na** ^**+**^ **/K** ^**+**^ **-ATPase α3 subunit knockdown sensitized T98G cells to TMZ.** To test the hypothesis that the high expression of Na^+^/K^+^-ATPase, especially its α3 subunit, plays a critical role in drug resistance of glioblastoma cells, we targeted the α3 subunit using the RNAi technique to downregulate its expression in T98G cells. **A**. RT-PCR assay verified that the α3 subunit was knocked down by the α3 stealth RNAi in T98G cells. **B**. Trypan blue cell death assay showed that, compared to control (blank bars), α3 knockdown (filled bars) in the drug-resistant T98G cells induced a trend of increased cell death even without drug insult. The addition of TMZ (100 μM) for 48 hrs more than doubled cell death in α3 knockdown T98g cells compared to the TMZ-induced cell death without α3 knockdown. N = 3 for each group. * *P* < 0.05 vs. controls; ^#^ *P* < 0.05 vs. TMZ treatment in control cells. **C**. Western blots of AIF, cytochrome c, β-actin and VDAC in cytosolic and mitochondrial fractions of transfection vehicle control (lipofactamine) and α3 knockdown cells with or without TMZ. There was no noticeable difference in AIF expression under different conditions while TMZ appeared to affect cytochrome c levels in the cytosol and mitochondrial compartments. **D**. Quantitative analysis of the Western blotting showed that α3 knockdown drastically increased cytochrome c release into the cytosol, while the mitochondrial cytochrome c level decreased in TMZ-treated α3-knockdown cells. N = 3 independent assays in each group. * *P* < 0.05 vs. α3 knockdown controls.

Due to the marked high expression of the Na^+^/K^+^-ATPase α3 subunit in T98G cells compared to LN229 and astrocytes, we tested a possible relationship between Na^+^/K^+^-ATPase and TMZ sensitivity. To knockdown the Na^+^/K^+^-ATPase α3 subunit in T98G cells, cells were treated with stealth RNAi for 48 hrs to reduce the α3 subunit (Figure 7A). When tested in α3 subunit knockdown T98G cells, TMZ (100 μM, 48 hrs) caused significantly more cell death compared to control T98G cells or α3 knockdown T98G cells without TMZ exposure (Figure 7B). Western blot analysis showed that down regulation of the α3 subunit augmented cytochrome C release from mitochondria to the cytoplasm when cells were treated with TMZ (Figure 7C and D). On the other hand, translocation of AIF was not affected by this knockdown (Figure 7C).

## Discussion

The present investigation shows for the first time in cancer cells that blocking or down regulation of Na^+^/K^+^-ATPase induces a cell death phenotype that has characteristics of both apoptosis and necrosis. We show that disruption of K^+^ homeostasis is a key factor in the induction of apoptosis in human glioblastoma cells. Contrary to what is widely believed that a cell may either die from apoptosis or necrosis, ouabain induced cell death does not have typical features of apoptosis or necrosis. Although strong apoptotic features such as phosphatidylserine translocation, caspase activation and Bcl-2 reduction were detected, ouabain-induced cell death in these cells exhibited necrotic features as well, including cell swelling, mitochondrial injury, [Ca^2+^]_i_ increase, deteriorated cellular organelles and breakdown of the plasma membrane. Consistent with the multifaceted ionic changes, ultrastructural alterations include both necrotic and apoptotic features. Since much higher expression of the Na^+^/K^+^-ATPase α2/α3 subunits exists in drug-resistant glioblastoma cells compared to drug-sensitive and normal human glial cells, our data indicate that the α2 and/or α3 subunits are potential targets for anti-cancer treatments. This was demonstrated by the different ouabain induced dose-responses of TMZ-sensitive LN229 cells, TMZ-resistant T98G cells and normal human astrocytes. This principle was also specifically demonstrated in the subunit knockdown experiment. Furthermore, the hybrid cell death mechanism of multiple targets helped to overcome the TMZ resistance of glioblastoma cells. Taken together, this investigation provides a better understanding of the ionic and cellular mechanisms underlying ouabain-induced cell death in human glioblastoma cells and suggests a potential therapeutic target for glioblastoma treatment.

We noticed that ouabain-induced apoptotic changes in LN229 cells were not typical of those caused after valinomycin exposure. For example, valinomycin caused gradual and progressive cell volume shrinkage while ouabain did not show the same volume change. Instead of apoptotic cell shrinkage, ouabain causes an initial cell swelling followed by a gradual decrease in cell volume. According to our previous research in non-cancerous cells and the data in this investigation, the initial cell swelling is attributable to intracellular Na^+^ and Ca^2+^ accumulation, while the gradual volume decrease is associated with the slower process of intracellular K^+^ depletion [36]. The early cell swelling followed by cell volume reduction during ouabain exposure was analogous to the hybrid cell death model (concurrent apoptosis and necrosis in the same cell) that we reported before in cortical neurons [41]. The acute increase in Ca^2+^/Na^+^ accompanied with a gradual K^+^ depletion in glioblastoma cells are consistent with the unique time-dependent cell volume alterations. The ionic disruptions also can be linked to necrotic and apoptotic events in these cells.

Although extensive research has been focused on Na^+^/K^+^-ATPase in tumor cells in the past few years, virtually all investigations assume apoptosis is the underlying mechanism for its anti-cancer effect. In the effort to identify therapeutic targets, many studies have focused on the α1 subunit while only very few reports have looked at the role of the α3 subunit [50]. To understand the ionic mechanism mediating the anti-cancer property of cardiac glycosides, many research reports examined intracellular Na^+^ accumulation and the Na^+^-dependent Ca^2+^ increases (e.g. Ca^2+^ oscillations) via enhanced reverse operation of the Na^+^/Ca^2+^ exchanger [31–33]. This research focus, however, overlooks the most abundant intracellular cation K^+^ and disregards the K^+^ role in apoptotic cell volume regulation and in the induction of apoptotic cascade. This was most likely due to the influence of many early investigations that simply linked K^+^ gradient to membrane potential regulation and the consequent influence on Ca^2+^ influx [42]. Accumulating evidence in recent years, however, has endorsed that a pro-apoptotic K^+^ efflux is an integrated cellular event in the early stage of apoptosis in non-cancerous neuronal, glial and peripheral cells [34–36]. We now show new evidence that this K^+^-mediated apoptotic mechanism similarly takes place in glioblastoma cells.

In the present investigation, low concentrations of ouabain and the induced hybrid cell death mechanism effectively sensitize drug-resistant glioblastoma cells to the anti-cancer effect of TMZ. It is important to note that, in this anti-cancer strategy, only a low concentration of ouabain is needed to sensitize the drug-resistant cancer cells. At the level of Na^+^/K^+^ pump down-regulation, survival of normal neuronal cells or even drug-sensitive glioblastoma cells are not affected. This is most likely due to the fact that expression of Na^+^/K^+^-ATPase is selectively and markedly higher in drug-resistant cells as shown in T98G cells. This selectivity supports a possible clinical significance of targeting Na^+^/K^+^-ATPase in glioblastoma treatment. Moreover, ouabain can pass through the blood brain barrier [51], which facilitates clinical applications of ouabain-like drugs in the potential treatment for brain tumors.

We previously showed that the sublethal low concentration of ouabain (0.1 μM) could markedly enhance the vulnerability of neuronal cells to pathological insults of glutamate, ceramide and β-amyloid [43]. The present study further suggests that ouabain and likely other cardiac glycosides can sensitize glioblastoma cells to conventional chemotherapy drugs via activation of multiple cell death mechanisms. While most cytotoxic anticancer drugs including alkylating agents such as TMZ have been shown to induce apoptosis [52], cancer cells could potentially be targeted by other death mechanisms such as necrosis, autophagy, senescence, or mitotic catastrophe [53]. To overcome the resistance of glioblastoma cells to TMZ, it has been postulated that it is possible to combine TMZ with other drugs to enhance glioblastoma cell response to TMZ cytotoxicity. In a randomized and double-blind trial for glioblastoma multiforme, adding chloroquine to conventional treatment including TMZ showed mediocre results, probably because of the small sample size [54]. Other combination regimens include adding epigallocatechin gallate (EGCG) and oxygen-diffusion enhancing trans-sodium crocetinate (TSC); the later is now in phase I/II clinical trials [55, 56].

In recent years, the mixed form of cell death of similar or partly overlapped definitions have been reported and are given different names such as aponecrosis and necroptosis [57–59]. We prefer the term hybrid death since it covers different known and novel cell death mechanisms. While the results from this investigation are encouraging, we acknowledge the need for further experiments to fully characterize the nature of cell death and delineate the detail dose–response relationship of Na^+^/K^+^-ATPase activity and viability of glioblastoma cells. As an *in vitro* investigation, our data provide some initial mechanistic information. The in vivo verification of the mechanism in an anti-cancer therapy will be essential. In addition to ouabain, application of other cardiac glycosides in the anti-cancer treatment should be tested since these clinical drugs have been used for the treatment of congestive heart failure and cardiac arrhythmia for many years. In addition to its effect on ionic homeostasis, Na^+^/K^+^-ATPase has been proposed to have a distinct function of directly regulating a number of intracellular signaling pathways [60]. Regulation of these signals may have important impacts on cell viability status and contribute to the anti-cancer effect of Na^+^/K^+^-ATPase inhibition. For example, it will be interesting to investigate the role of the PI3K/Akt/mTOR pathway that is regulated by Na^+^/K^+^-ATPase and which plays a major role in cancer cell survival [61].

## Conclusion

In using ouabain as a representative Na^+^/K^+^-ATPase inhibitor, this study demonstrates the induction of hybrid cell death in glioblastoma cells and enhanced cell death of a TMZ-resistant cancer cell line. Based on its high expression level in TMZ-resistance cells, Na^+^/K^+^-ATPase may be a therapeutic target for the treatment of glioblastoma and sensitizing glioblastoma cells to conventional chemotherapy.

## Authors’ information

SY is the O. Wayne Rollins Professor in the Department of Anesthesiology and a Professor in the Department of Hematology and Oncology, Emory University School of Medicine.

## Electronic supplementary material

Additional file 1: Figure S1: Valinomycin-induced apoptotic death of LN229 cells. Western blotting and immunohistochemistry were applied to examine the effect of valinomycin on LN229 cells. **A**. Western blots of Bcl-2, caspase-3 and caspase-9 at 6, 12 and 24 hrs after valinomycin (10 μM) treatment. **B** to **D**. Valinomycin treatment significantly increased caspase-3 and caspase-9 expression and reduced the anti-apoptotic protein Bcl-2 expression in LN229 treated cells. N = 3 in each group. * *P* < 0.05 vs. vehicle control (CTL). **E**. Immunohistochemical staining for apoptosis inducing factor (red) and nuclei (Hoechst, blue) showed that valinomycin induced AIF translocation from cytoplasm to nucleus 6 hrs after valinomycin treatment. (TIF 485 KB)

Additional file 2: Figure S2: Ouabain-induced cellular Na^+^ and Ca^2+^ changes in LN229 cells. Intracellular Na^+^ and Ca^2+^ was assessed in LN229 cells using the Na^+^ dye SBFI-AM and Ca^2+^ fluorescent dye Fluo-4 AM, respectively. **A** and **B**. SBFI-AM fluorescent imaging showed a gradual increase in the intracellular Na^+^ content. B is the quantified analysis of Na^+^ imaging showing ouabain-induced acute increase in intracellular Na^+^ content of LN229 cells within 5–60 min after application of ouabain. **C**. Fluo-4-AM imaging detected [Ca^2+^]_i_ increases in LN229 cells 1 to 3 hrs after application of 1 μM ouabain. Co-applied BAPTA-AM (1 μM) effectively prevented the [Ca^2+^]_i_ change. **D**. The intensity of Fluo-4 fluorescence was quantified using Image J software (NIH). Ouabain doubled the [Ca^2+^]_i_ at 3 hrs after exposure and the Ca^2+^ level was gradually subsided. N = 240 cells from 3 independent assays. *p < 0.01 vs. time 0. **E**. MTT assay showed that although oubain (1 μM, 24 hrs) reduced cell viability of LN229 cells, the addition of BAPTA-AM did not show a significant protection on ouabain-induced cell death. As an intracellular Ca^2+^ chelator, BAPTA-AM alone showed some toxicity to LN229 cells. N = 3 independent assays. * *P* < 0.01 vs. control. (TIF 709 KB)

## Abbreviations

GBM: Glioblastoma multiforme
TMZ: Temozolomide
WHO: World Health Organization
CNS: Central nervous system
FBS: Fetal bovine serum
MTT: 3-(4,5-dimethyl-thiazol-2-yl)-2,5-diphenyltetrazolium
PS: Phosphatidylserine
PBS: Phosphate-buffered saline
TMRM: Tetramethylrhodamine methyl ester
AIF: Apoptosis-inducing factor
EGCG: Epigallocatechin gallate
TSC: Trans-sodium crocetinate.
