# Supplementary figures and images for: Inhibition of Na+/K+-ATPase induces hybrid cell death and enhanced sensitivity to chemotherapy in human glioblastoma cells
Source: BMC Cancer. 2014 Sep 26;14:716. doi: 10.1186/1471-2407-14-716 (PMC4190379; doi:10.1186/1471-2407-14-716)

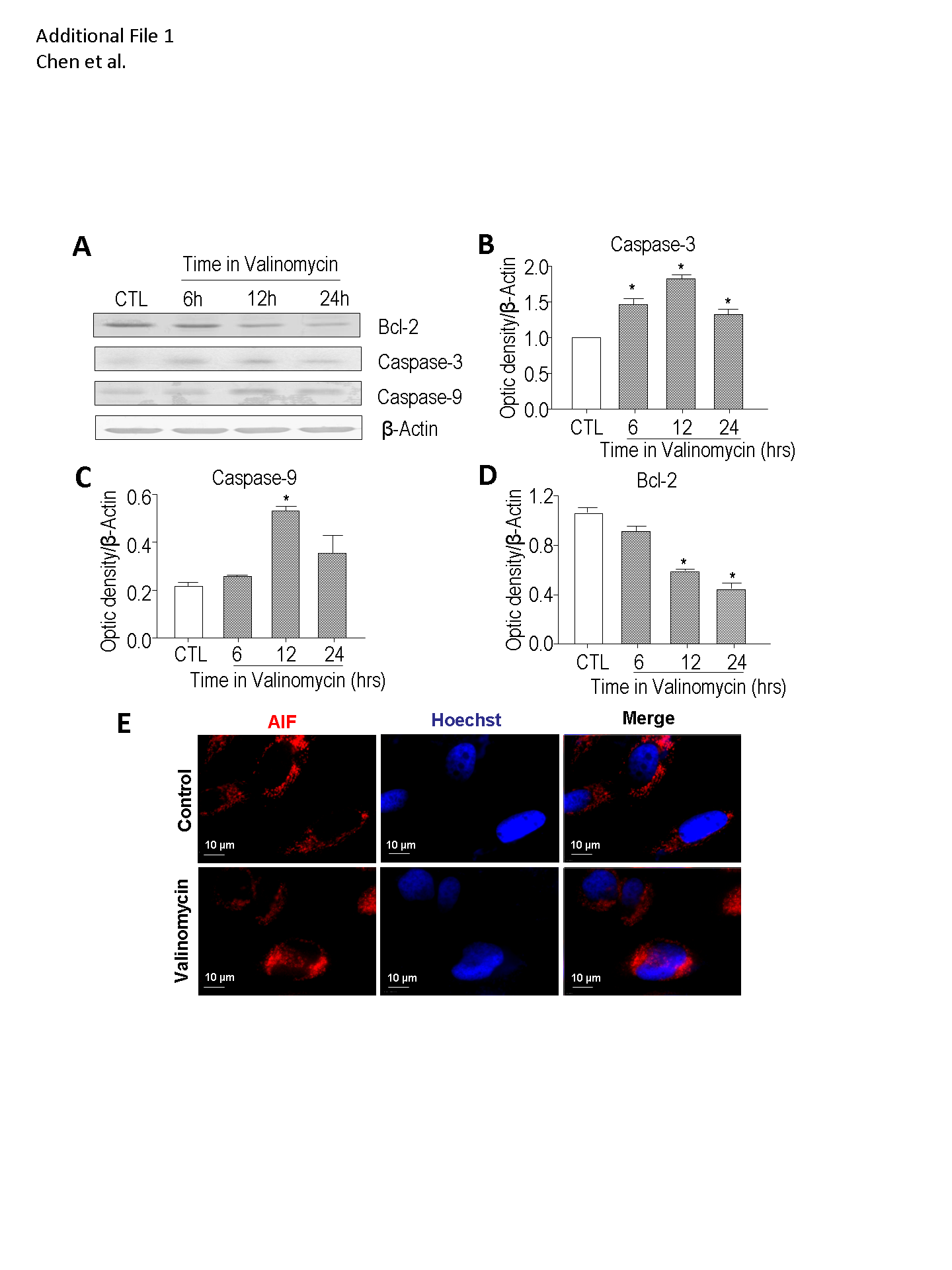

Supplement: Supplementary file 1 — Additional file 1: Figure S1: Valinomycin-induced apoptotic death of LN229 cells. Western blotting and immunohistochemistry were applied to examine the effect of valinomycin on LN229 cells. A. Western blots of Bcl-2, caspase-3 and caspase-9 at 6, 12 and 24 hrs after valinomycin (10 μM) treatment. B to D. Valinomycin treatment significantly increased caspase-3 and caspase-9 expression and reduced the anti-apoptotic protein Bcl-2 expression in LN229 treated cells. N = 3 in each group. * P < 0.05 vs. vehicle control (CTL). E. Immunohistochemical staining for apoptosis inducing factor (red) and nuclei (Hoechst, blue) showed that valinomycin induced AIF translocation from cytoplasm to nucleus 6 hrs after valinomycin treatment. (TIF 485 KB) [file 12885_2014_4905_MOESM1_ESM.tif]

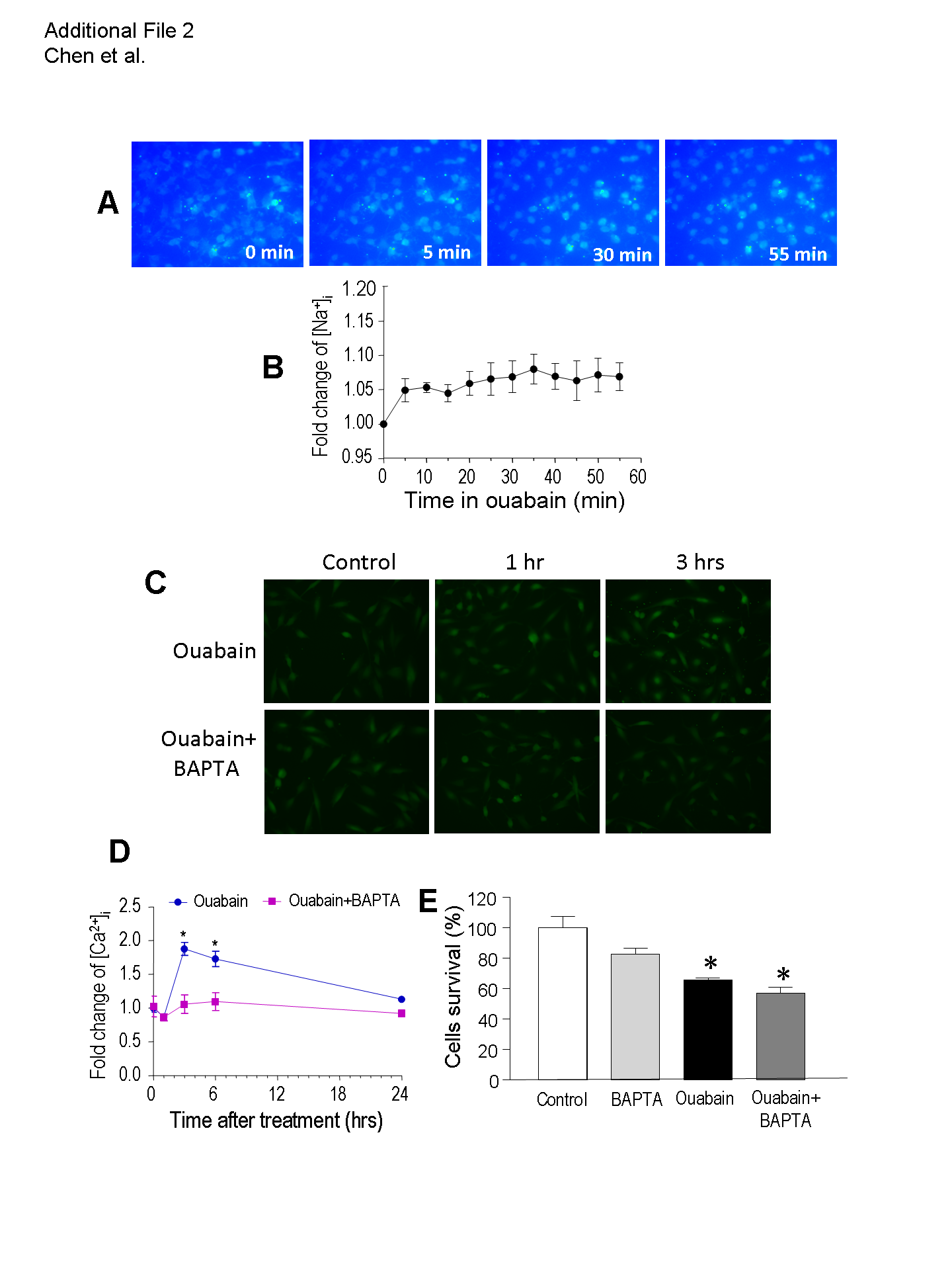

Supplement: Supplementary file 2 — Additional file 2: Figure S2: Ouabain-induced cellular Na+ and Ca2+ changes in LN229 cells. Intracellular Na+ and Ca2+ was assessed in LN229 cells using the Na+ dye SBFI-AM and Ca2+ fluorescent dye Fluo-4 AM, respectively. A and B. SBFI-AM fluorescent imaging showed a gradual increase in the intracellular Na+ content. B is the quantified analysis of Na+ imaging showing ouabain-induced acute increase in intracellular Na+ content of LN229 cells within 5–60 min after application of ouabain. C. Fluo-4-AM imaging detected [Ca2+]i increases in LN229 cells 1 to 3 hrs after application of 1 μM ouabain. Co-applied BAPTA-AM (1 μM) effectively prevented the [Ca2+]i change. D. The intensity of Fluo-4 fluorescence was quantified using Image J software (NIH). Ouabain doubled the [Ca2+]i at 3 hrs after exposure and the Ca2+ level was gradually subsided. N = 240 cells from 3 independent assays. *p < 0.01 vs. time 0. E. MTT assay showed that although oubain (1 μM, 24 hrs) reduced cell viability of LN229 cells, the addition of BAPTA-AM did not show a significant protection on ouabain-induced cell death. As an intracellular Ca2+ chelator, BAPTA-AM alone showed some toxicity to LN229 cells. N = 3 independent assays. * P < 0.01 vs. control. (TIF 709 KB) [file 12885_2014_4905_MOESM2_ESM.tif]
